# Examination survey on health and nutrition of the adult population in Germany between 2019 and 2021 – the current status of planning

**DOI:** 10.17886/RKI-GBE-2018-050

**Published:** 2018-05-16

**Authors:** Antje Gößwald

**Affiliations:** Robert Koch Institute, Berlin, Department of Epidemiology and Health Monitoring

The Robert Koch Institute (RKI) and the Max Rubner Institute (MRI) are jointly conducting a nationally representative study on the health and nutrition of the adult population in Germany. The aim of the study is to merge the health monitoring programme conducted at the RKI, the DEGS and GEDA studies, and nutrition monitoring with the National Food Consumption Study [1]. The new study will focus on physical and mental health, health-related behaviour, nutrition and nutrition-related behaviour, people’s environmental and living conditions, as well as their social situation and uptake of medical services. The study will also integrate the European Health Interview Survey.

The study aims to achieve a sample size of 12,500 participants, and will begin in spring 2019 and continue until summer of 2021. The target population is adults aged between 18 and 79 whose primary residence is registered in Germany. Initially, 300 sample points, stratified according to federal state and municipality type, are to be randomly selected. The participants will then be selected at random from the local population registers in the respective sample points.

Surveying will take place using a sequential mixed-mode design with web-based questionnaires and written questionnaires, and partly through computer-assisted personal interviews. This will include a 24-hour dietary recall of the participants’ current food intake and an interview on medication and nutritional supplements. The examination study will comprise numerous laboratory tests on blood and urine samples, anthropometry (height, weight and bioimpedance), resting blood pressure, pulmonary function, neurocognitive tests, physical function (strength, mobility and balance) and measurements of physical activity. The medical examinations are to be conducted in the selected locations in temporary mobile examination centres by teams with medical training.

Due to the fact that people are currently less willing to participate in population-based interview surveys and examinations, as well as societal changes caused by demographic changes, the aging of society and the influx of migrants, the methods that researchers use for participant acquisition need to be adapted regularly. The new survey, therefore, will include specific measures aimed at better reaching groups such as the elderly and people with a limited knowledge of German.

The examination survey on health and nutrition of the adult population in Germany will provide further nationwide representative data that enable estimates to be made of current prevalences and trends, as well as context analyses on issues related to public health.

## References

[1] Krems C Bauch A Götz A (2006) Methoden der Nationalen Verzehrsstudie II. Ernährungs-Umschau 53(6):44–50

